# Integrating Low-Stack Photonic Crystals with the Honeycomb-like Structural Framework to Enhance the Photovoltaic Performance in Perovskite Solar Cells

**DOI:** 10.1021/acsomega.3c09868

**Published:** 2024-02-15

**Authors:** Chen Yuan, Yibin Yang, Le Huang, Ye Xiao

**Affiliations:** School of Materials and Energy, Guangdong University of Technology, Guangzhou 510006, China

## Abstract

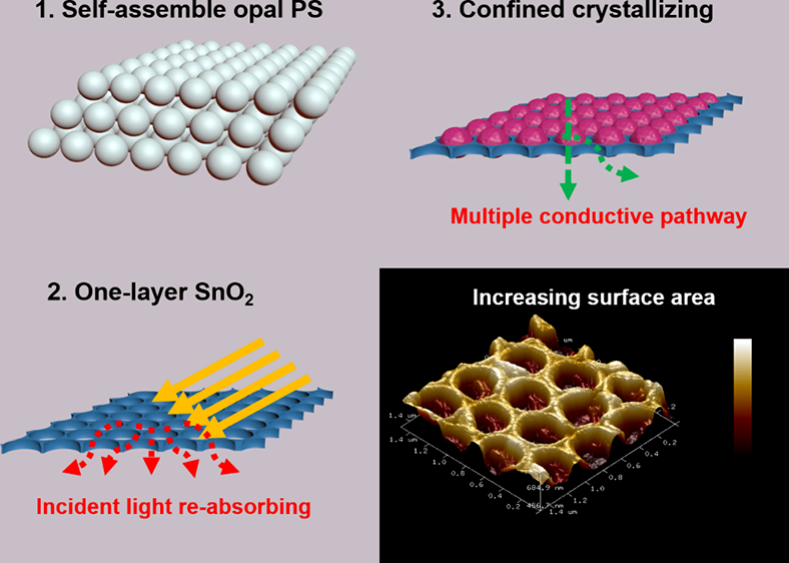

An inverse opal structure of SnO_2_ with a honeycomb morphology is introduced as the framework for the attached perovskite materials and functional layers in the hybrid perovskite-based solar cells simultaneously. Three different pore sizes of polystyrene microsphere layers, with diameters of 350, 480, and 600 nm, were fabricated through a vertical self-assembly vaporization technique. The polystyrene (PS) layer served as the sacrificial template for the inverse opal structure. By controlling the spinning parameters, the inverse opal-structured SnO_2_ layer was used to constrain them into a single-layer stacking structure. These layers with varying pore sizes were subsequently applied onto a dense electron transport layer that is in contact with the perovskite layer. A carbon electrode is used as photovoltaic solar cells. The major benefits of this approach were systematically analyzed through structural characterizations and various means. The semiphotonic crystal layer induces modulation effects, resulting in increased light absorption and surface area, which leads to a substantial increase in short-circuit density. By studying the electrochemical properties in the dark to exclude the influence of optical effects, we attribute the slight increase in the fill factor to the increased surface area, which enhances carrier transport. Among the different layers, the inverse opal layer prepared with 480 nm polystyrene microspheres displayed superior photovoltaic performance parameters due to its appropriate surface area and relatively higher light absorption. The power conversion efficiency of the MAPbI_3_ perovskite solar cell showed a relative enhancement of 55%. Additionally, aging tests demonstrated that devices with the additional structural layer exhibited good endurance under conventional atmospheric conditions after 1440 h of aging.

## 1 Introduction

Driven by the remarkable power conversion efficiency (PCE) achieved by hybrid organic–inorganic perovskite (HOIP)-based solar cells (PSCs), extensive research has been devoted to this field for decades.^1,2^ A typical configuration for PSCs consists of stacked layers,^3^ including a transparent conductive electrode (conductive glasses), and semiconductors with suitable band gaps serving as the compacted electron transfer layer (ETL),^4^ which also acts as a good scaffold for the attached optically active perovskite materials.^5^ This is followed by a hole transfer layer (HTL) composed of organic materials with suitable band alignment^6^ and a counter electrode (carbons or gold) to collect the output circuits.^7,8^ From the perspective of ETL scaffolds, SnO_2_ possesses unique advantages in the application of planar perovskite solar cells (PSCs) compared to other wide-gap semiconductors due to its excellent electronic properties,^9^ such as high charge mobility (240 cm^2^/(V s))^10^ and a wide band gap (3.6 eV)^11^ with proper band alignment with perovskites. These advantages in the electronic structure result in higher charge injection efficiency but only when the layer has a controlled thickness^12^ as longer transport lengths lead to energy loss. In terms of process compatibility, SnO_2_ in the colloidal precursor form can easily undergo diversified structural modulations, allowing for precise control of nanostructures.^13^ This enhances the interfacial contact and eliminates electronic trap states.^14^ Based on these considerations, we developed an inverse opal-structured SnO_2_ with a single-layered longitudinal periodicity through colloidal solution-based techniques. This unique morphology brings three major benefits to the photovoltaic performance of PSCs. First, the macroporous framework of the inverse structure can embed confined crystallized perovskites, providing more contact interfaces with photoactive materials.^15^ Second, by controlling the thickness of the inverse skeletons to several tens of nanometers, the embedded perovskite materials in the separated honeycomb structured cells form parallel conductive pathways, increasing the efficiency of carrier transport.^16^ Third, the one-layered inverse opal structure exhibits a semiphotonic crystal structure, further enhancing sunlight absorption^17^ and increasing the short-circuit density to some extent. Therefore, a systematic investigation was conducted by using different structural building blocks of SnO_2_ frameworks with varying diameters based on polystyrene (PS) microspheres. Opal structures with PS diameters of 350, 480, and 600 nm were initially formed using self-assembly techniques based on surface tension. Subsequently, the corresponding inverse opal SnO_2_ structures were sintered at a low synthesis temperature, and the MAPbI_3_-based perovskite materials were crystallized within the confined spaces of the individual honeycomb cells. Structural characterization, optical property characterization, and photovoltaic performance were systematically analyzed for several groups of samples. As a result, compared to the planar structure, the honeycomb structured matrix with interfacial engineering enhances electron transport, while the single-layered semiphotonic crystal structure enhances light absorption. Two mechanisms, one is the semiphotonic stop band and the other is the increased surface area for the attached perovskite materials, contribute to a significant increase in short-circuit current density (*J*_SC_) and a slight increase in the fill factor (FF) of the solar cells. Among the samples, the inverse opal SnO_2_ layer prepared by using 480 nm polystyrene microspheres exhibited the highest photovoltaic performance parameters. Ultimately, the average power conversion efficiency (PCE) of the MAPbI_3_-based PSCs increased from 7.51 to 11.67%, representing a relative improvement of 55% compared to that of the planar SnO_2_ control group. Furthermore, the modules retained 88% of the initial PCE efficiency after a 1440 h aging process, which is beneficial for further commercial applications.

## 2 Experimental Methods

### 2.1 Chemicals

Anhydrous ethanol (99%), acetone (99%), toluene (99%), and isopropyl alcohol (99%) were purchased from Guangzhou Chemical Reagent Factory. Ethyl acetate (99.5%), *N*,*N*-dimethylformamide (99.9%), and dimethyl sulfoxide (99.9%) were purchased from Aladdin. Tin(II) chloride dihydrate (98%) was purchased from Macklin. Lead iodide (99.9%) and methylammonium iodide (99.5%) were purchased from Xi’an Baolite. High-temperature carbon slurry with a surface resistivity of 20–30 Ω·sq^–1^ was purchased from Shanghai Maitewen. FTO conductive glass was purchased from Enbu, with a surface resistivity of 8 Ω·sq^–1^. All reagents were used as received from Maitewen without further purification. Ultrapure water was used for all experimental steps.

### 2.2 Precursor Solution Preparations

#### 2.2.1 Polystyrene (PS) Preparation

Deionized water (120 mL) was taken and placed in a three-necked flask and heated in a water bath to 85 °C. Separately, 10, 15, and 20 mL of PS solution were taken and placed in three-necked flasks and stirred at a speed of 300 r/min for 10 min. Potassium persulfate (0.1 g) was dissolved in 5 mL of deionized water, added to the three-necked flask, and continuously stirred at 300 r/min for 12 h, resulting in monodisperse PS microsphere solutions with particle sizes of 350, 480, and 600 nm. Deionized water (48 mL) and 2 mL of anhydrous ethanol were mixed, and 0.5 mL of the PS microsphere solution was added. The mixture was sonicated for 30 min to obtain a dispersed PS microsphere solution.

#### 2.2.2 SnO_2_ Precursor Preparation

Tin(II) chloride dihydrate (564 mg, SnCl_2_·2H_2_O) was dissolved in 25 mL of isopropanol to obtain a SnO_2_ precursor solution.

### 2.3 Device Fabrication

**Scheme 1:**
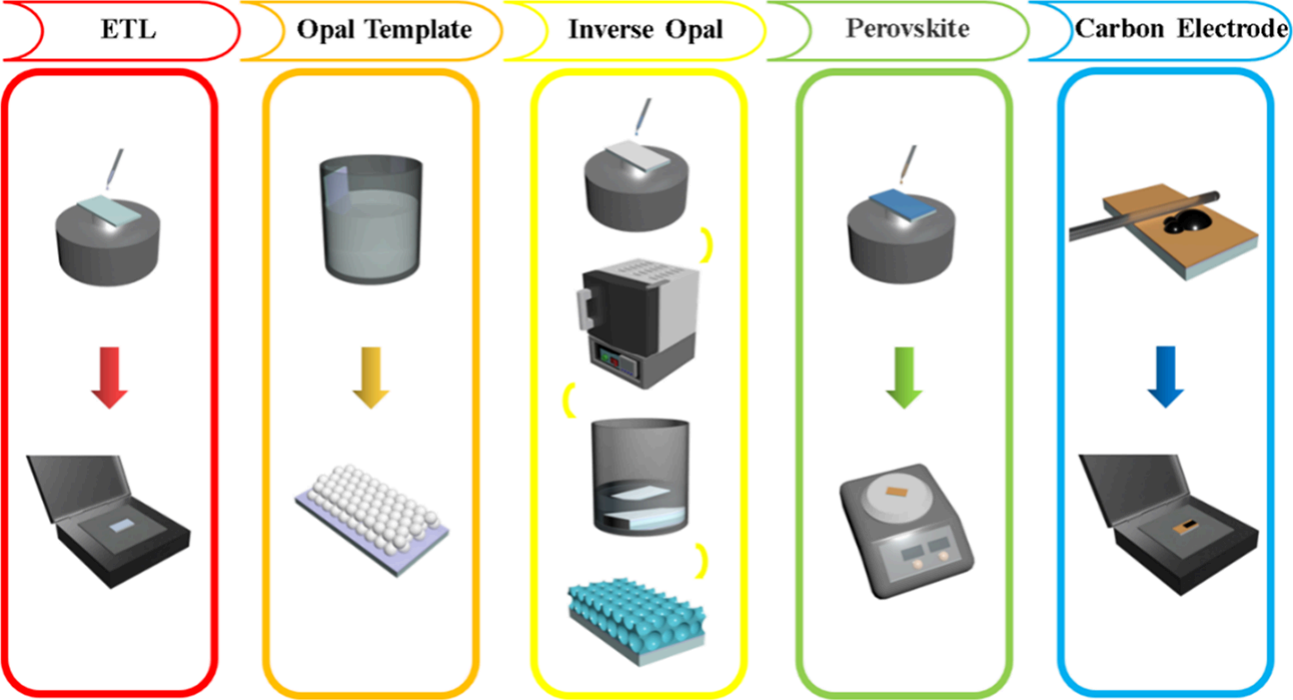
Synthesizing Process of the Different Layers and Devices

Each layer of the device is fabricated as an illustration of the above Scheme 1 diagram:

1. Planar SnO_2_ ETL fabrication: The FTO conductive glass was sequentially cleaned in acetone, anhydrous ethanol, and deionized water by ultrasonication for 25 min. A layer of SnO_2_ precursor solution was spin-coated on the FTO conductive glass at a speed of 3000 rpm for 30 s followed by annealing at 180 °C for 90 min to obtain the Flat-SnO_2_ layer.
2. Opal PS template fabrication: The Flat-SnO_2_ layer was vertically immersed in the PS microsphere-dispersed solutions with different particle sizes, and a small amount of sodium dodecyl sulfate (SDS) was added to the dispersed solutions. The samples were placed in a convection drying oven and heated at 60 °C for 12 h to form the PS microsphere inverse layer.
3. Inverse opal SnO_2_ layer fabrication: A layer of SnO_2_ precursor solution was spin-coated on the inverse opal layer at a speed of 2000 rpm for 60 s. The spin-coating process was repeated three times to ensure complete infiltration of the solution into the template. After spin-coating, the samples were annealed in a muffle furnace at a heating rate of 1 °C/min to 180 °C for 90 min. After cooling to room temperature, the samples were immersed in toluene, acetone, anhydrous ethanol, and deionized water for 30 min to completely remove the PS microspheres, resulting in PS350-SnO_2_, PS480-SnO_2_, and PS600-SnO_2_ layers.
4. MAPbI_3_ layer fabrication: 0.461 g of PbI_2_ and 0.159 g of MAI were dissolved in a mixture solution of 0.662 mL of DMF and 0.078 mL of DMSO to obtain the MAPbI_3_ precursor solution. The MAPbI_3_ precursor solution was spin-coated onto the PS350-SnO_2_, PS480-SnO_2_, PS600-SnO_2_, and Flat-SnO_2_ layers at a speed of 4000 rpm for 25 s. After spinning for 7 s, 0.1 mL of ethyl acetate was quickly dropped into the precursor solution. After spin-coating, the samples were heated at 100 °C for 10 min.
5. Photovoltaic device assembling: the carbon electrode was directly deposited on the MAPbI_3_ layer using a doctor-blade coating method and heated at 100 °C for 20 min to form the carbon electrode.

### 2.4 Characterization

Cross-sectional and top-view images of the perovskite solar cell layers were observed using field-emission scanning electron microscopy (FE-SEM, SU8010, Hitachi, Tokyo, Japan). The transmittance, absorbance, and diffuse reflectance of the inverse opal SnO_2_ layer and Flat-SnO_2_ layer were characterized by using a UV-visible spectrophotometer (UV-3600 Plus). The structures of the electron transport layer and the perovskite layer were determined using a multifunctional X-ray diffractometer (XRD) system (D/MAX Ultima IV, Rigaku). The current density–voltage (*J*–*V*) curves of the PSCs were measured using a Keithley 2400 digital source meter and a solar simulator (Newport Oriel 94043A) under an incident light power of 100 mw/cm^2^ and AM 1.5 G spectrum. The effective illuminated area was 0.09 cm^2^. Electrochemical impedance spectroscopy was performed using an electrochemical workstation, and the equivalent circuit model was fitted using ZSimpWin. The atomic force microscopy (AFM) data was collected by Dimension FastScan from Bruker.

## 3 Results and Discussion

**Figure 1:**
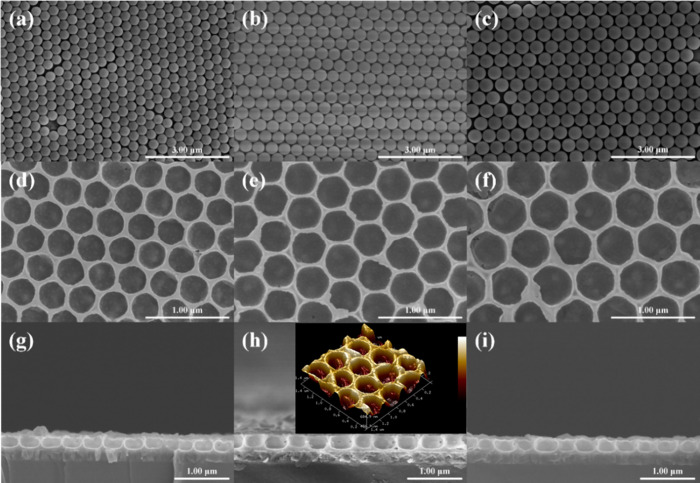
SEM top-view images of polystyrene (PS) microspheres inverse opal with particle sizes of (a) 350 nm, (b) 480 nm, and (c) 600 nm; top-view SEM micrographs of the inverse opal layers for (d) PS350-SnO_2_, (e) PS480-SnO_2_, and (f) PS600-SnO_2_; cross-sectional SEM micrographs for (g) PS350-SnO_2_, (h) PS480-SnO_2_, and (i) PS600-SnO_2_.

The preparation of monolayer protein crystals using polystyrene (PS) microspheres was investigated using SEM. Through the self-assembly formation technique under a controlled evaporation process, these microspheres orderly stack in a face-centered cubic (fcc) structure with closely packed (111) planes on the flat SnO_2_ surface. The top-view images in Figure 1a–c reveal the PS microspheres with diameters of 350, 480, and 600 nm, respectively. It can be concluded that all the PS microspheres exhibited a uniform size distribution without distortion under negligible stress as they were arranged in only one layer in the longitudinal direction. To create the inverse opal structure, the opal layer was used as a sacrificial template. The residual space, filled with air, comprised the opal inverse structure. The SnO_2_ precursor solution permeated the interstices of the opal structure and then sintered to form a solid framework. As shown in Figure 1d–f, the inverse opal framework exhibits a dense packing morphology composed of units with a honeycomb-like structure. The pore sizes of the honeycombs are 320, 410, and 480 nm, respectively, which are slightly smaller than the corresponding initial diameter of the PS microspheres.^18^ The views in Figure 1g–i show a cross-sectional view of the inverse opal SnO_2_ layer. The bowl-shaped structures observed are approximately half the height of one unit, providing further evidence of a single-layer stacked structure originating from the initial opal layer. A clearer 3D view of the PS480 inverse layer is shown in the inset of Figure 1h by AFM instruments, and the large view is given in Figure S2. The rough surface of the framework provides good nucleation sites for the attached perovskite materials. Thus, the techniques for controlling the spinning speed successfully resulted in a structure with controlled counts of layers. Notably, it was observed that there were protrusions on the inverse layer, indicating the presence of defects in the formation process of the inverse opal layer. The formation of these defects could be mainly attributed to two issues; one is the adhesion of precursor solution with a higher-than-average liquid level on the PS microspheres during the spinning process; the other is the characteristics of the condensation reaction for the SnO_2_ precursor. During the sintering process, the precursor becomes denser. As the structural fluctuations in the periodic structure may deteriorate the light reflectance spectrum around the photonic stop band, the superb high reflectance spectrum around the photonic stop band is compromised.^19^ Unfortunately, it is not possible to create a perfect one-layer photonic crystal with limited resources. However, the increased surface area and the honeycomb cells can also enhance the photovoltaic performance of devices indeed.^20^ The optimization of the preparation process is crucial for further applications in various related research fields.

**Figure 2:**
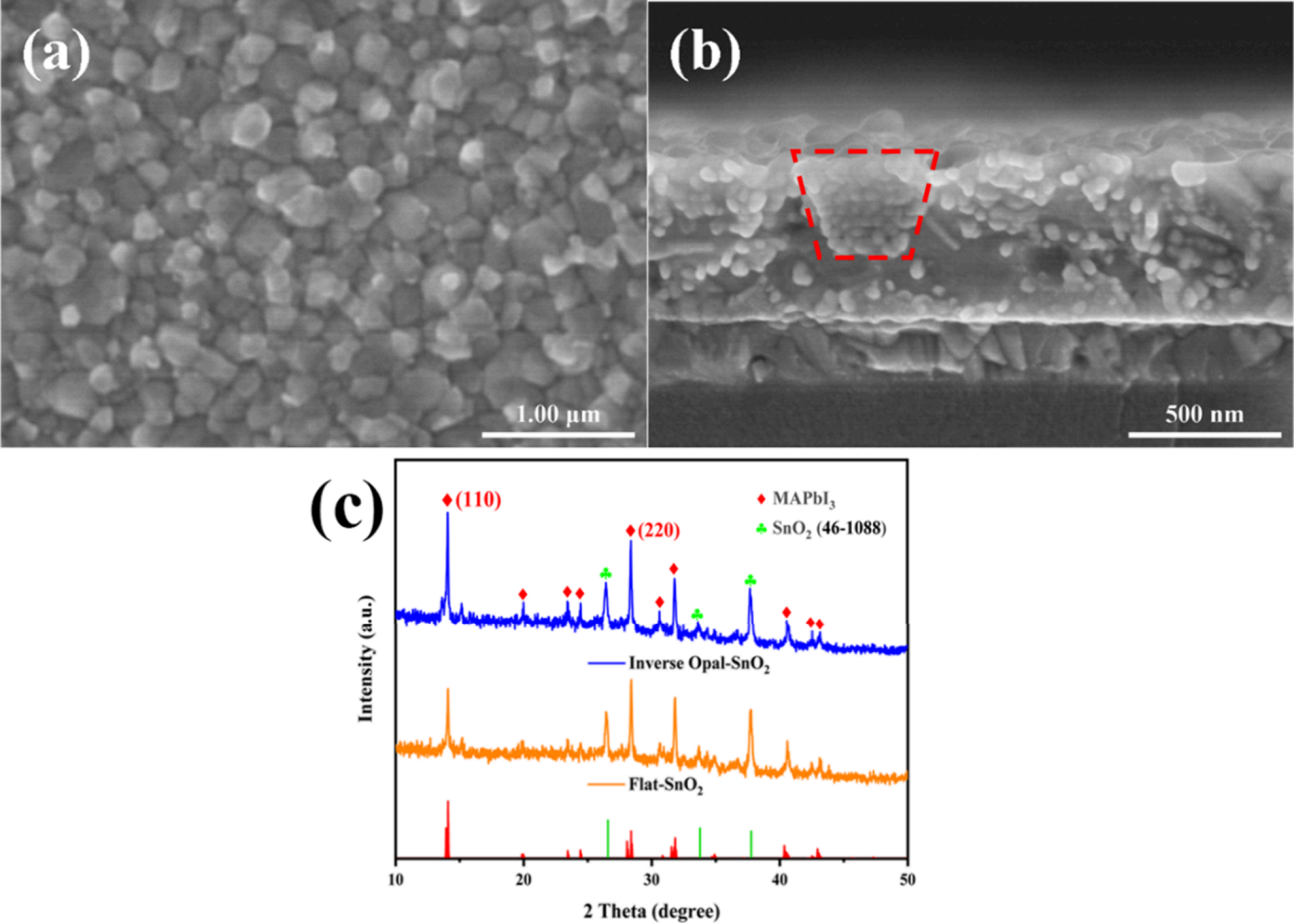
SEM top-view (a) and cross-sectional view (b) of the MAPbI_3_ perovskite layer on the inverse opal layer and (c) XRD spectra for MAPbI_3_ on Flat-SnO_2_ and inverse opal SnO_2_.

Figure 2a and Figure 2b show the SEM top-view and cross-sectional view of the MAPbI_3_ perovskite layer on the inverse opal layer, respectively. As shown in Figure 2a, the perovskite particles of MAPbI_3_ are relatively uniform in size, completely covering the inverse opal layer, and no noticeable surface pinholes were observed. As shown in Figure 2b, the fine crystalline structure of the MAPbI_3_ perovskite fills the concave space of the honeycomb cell, which is depicted in the red-dashed trapezoid. The two layers are well connected in the longitudinal direction and transverse direction with no gaps formed between the perovskite layer and the electron transport layer. Therefore, the electrical circuit, which is generated from the perovskite layer, would have two different pathways to conduct and then reduce the electrical resistance. What is more, the thickness of the MAPbI_3_ perovskite layer is approximately 530 nm, which is sufficient for the absorption coefficient of MAPbI_3_. The XRD patterns of the MAPbI_3_ layers fabricated on the PS480-SnO_2_ layer and the Flat-SnO_2_ layer are shown in Figure 2c, revealing the two main phases, MAPbI_3_ (theoretically computed) and orthorhombic SnO_2_ phases as the inverse structure (PDF #46-1088). It can be observed that compared to the Flat-SnO_2_ layer, the intensity of each peak corresponding to MAPbI_3_ is slightly enhanced on the PS480-SnO_2_ layer. Additionally, the peak intensity corresponding to the (110) crystal plane of MAPbI_3_ exceeds that of the other higher index crystal plane of (220). This preferential orientation may be attributed to the favorable nucleation sites provided by the inverse opal structure, which is beneficial for improving the overall performance of MAPbI_3_ perovskite solar cells.^21,22^

**Figure 3:**
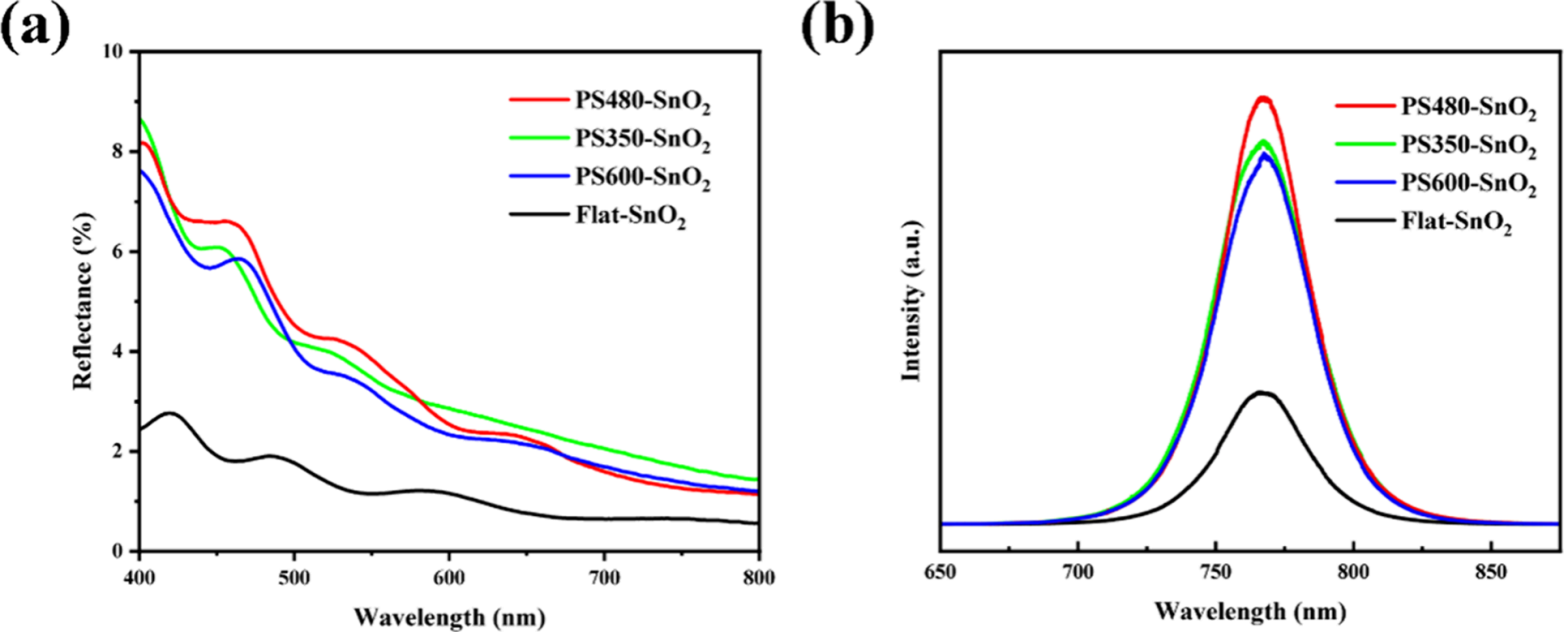
(a) UV-visible diffuse reflectance spectra of PS350-SnO_2_, PS480-SnO_2_, PS600-SnO_2_, and Flat-SnO_2_ layers; (b) photoluminescence spectra of MAPbI_3_ layers fabricated on PS350-SnO_2_, PS480-SnO_2_, PS600-SnO_2_, and Flat-SnO_2_ layers under excitation of a 532 nm laser.

As the inverse opal layer indeed has a long-range ordered periodic structure, when neglecting minor defects and protrusions, the Bragg reflection phenomenon becomes significant for this semiphotonic crystal structure, and the optical properties of the inverse opal SnO_2_ layer become different. As shown in Figure S1a, the color change of the inverse opal can be clearly observed by bare eyes under sunlight illumination by rotating the angle.^23^ In contrast, the flat sample in Figure S1b shows a single color. According to the UV-visible diffuse reflectance spectra in Figure 3a, the reflectance in the visible range of PS350-SnO_2_, PS480-SnO_2_, and PS600-SnO_2_ layers is higher than that of the Flat-SnO_2_ layer. Moreover, it is observed that in the 400–600 nm range, the reflectivity of the PS480-SnO_2_ layer is higher than those of the PS350-SnO_2_ and PS600-SnO_2_ layers. In other words, the inverse opal layer could enhance the secondary utilization of sunlight by reabsorbing the unabsorbed energy flux that escapes from the photoactive layer above.^24^ Therefore, after depositing MAPbI_3_ layers on the inverse opal layers using a one-step antisolvent method, more photons should be absorbed compared to those on the Flat-SnO_2_ layer. As evidenced by the photoluminescence spectra in Figure 3b, there is a noticeable increase in the peak intensity around the exciton energy level for MAPbI_3_.^25^ Additionally, the PS480-SnO_2_ layer shows the highest PL peak intensity, which correlates well with its highest reflectivity.

**Figure 4:**
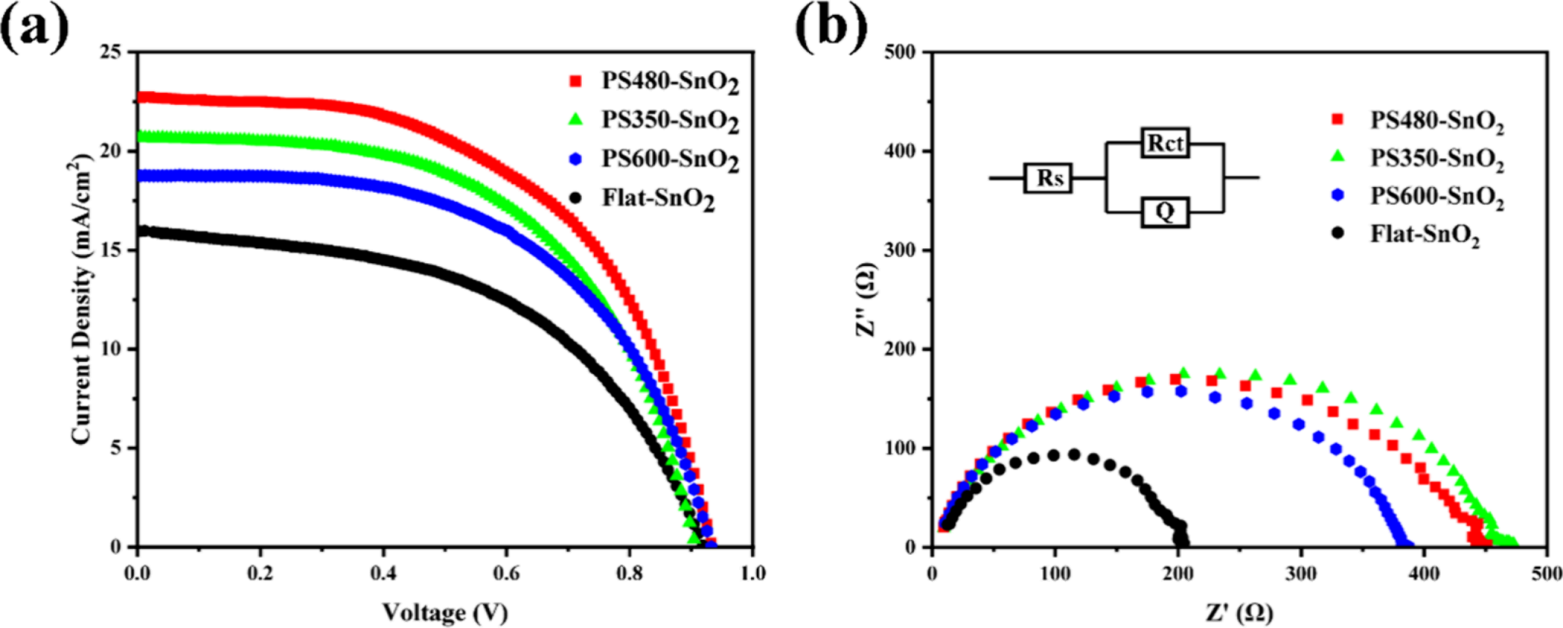
(a) *J*–*V* curves of MAPbI_3_ perovskite solar cells fabricated on PS350-SnO_2_, PS480-SnO_2_, PS600-SnO_2_ and Flat-SnO_2_ layers; (b) electrochemical impedance spectroscopy (EIS) spectra of MAPbI_3_ perovskite solar cells.

The photovoltaic performance of PV devices has been evaluated. As shown in Figure 4a, the *J*–*V* curves of MAPbI_3_ perovskite solar cells fabricated on Flat-SnO_2_, PS350-SnO_2_, PS480-SnO_2_, and PS600-SnO_2_ layers were investigated. Compared to Flat-SnO_2_, the solar cells with an inverse opal structure exhibited significantly improved *J*_SC_, ranging from 15.95 mA/cm^2^ to the highest value of 22.71 mA/cm^2^ for the PS480-SnO_2_ sample. Table 1 shows that the filing factor (FF) was also slightly increased, resulting in an overall enhancement of PCE of the inverse perovskite solar cells from 7.51 to 11.67%, representing a relative improvement of 55%. As mentioned before, there are three major benefits of the inverse opal framework. First, it induces light modulations through the photonic crystal by introducing a layer of periodic Bragg scattering within the structure.^26^ This increases the absorption of visible light by the solar cells. Second, the framework has a macroporous structure that provides a surface area approximately 3 times larger than flat SnO_2_.^17^ This, combined with the separated honeycomb structured cell, is the third benefit; the honeycomb structure creates parallel conductive pathways, which enhances the efficiency of carrier transport. Both mechanisms from optic properties and the porous morphology together contributed to enhanced *J*_SC_, and the sole characters of the porous morphology mainly contributed to the slightly increased FF as the framework facilitates faster extraction of photogenerated electrons from the perovskite interface, thus reducing the recombination of photogenerated electrons and holes. The further increased radius of the initial PS microspheres decreases the PCE performance of PS600 devices, which could be attributed to its decreased specific surface area as a too-large radius of units.

**Table 1:** Performance Parameters Obtained from the Measurement of *J*–*V* Curves for MAPbI_3_ Perovskite Solar Cells Based on Different Inverse Electron Transport Layers

	*V*_OC_ (V)	*J*_SC_(mA/cm^2^)	FF (%)	PCE (%)
Flat-SnO_2_	0.922	15.95	0.51	7.51
PS350-SnO_2_	0.908	20.70	0.57	10.63
PS480-SnO_2_	0.936	22.71	0.55	11.67
PS600-SnO_2_	0.936	18.77	0.56	9.79

To investigate the sole mechanisms induced by the increased surface area and morphology of honeycomb units, the EIS spectra for the carrier transport studies without incident light are shown in Figure 4b. As seen from the Nyquist plots of the four devices measured in the dark, the simplified equivalent circuit model of the Nyquist plot is also given in the inset of Figure 4b. This model consists of two resistances (*R*_s_ and *R*_ct_) and a constant phase element (*Q*). *R*_s_ corresponds to the series resistance of the device, while *R*_ct_ corresponds to the charge transfer resistance at the interface of the device.^27^ Due to the inverse opal structure, the modulation effect of light is lost. As a result, the device is influenced only by the specific surface area of the electron transport layer. With the increase in the specific surface area, the corresponding charge transfer resistance also increases. According to the fitted data (Table 2), the *R*_ct_ of the inverse opal devices is higher than that of the planar devices first. This indicates that the high specific surface area of a porous structure improves the speed of electron extraction and transport and inhibits the recombination of photogenerated electrons and holes. With a further increase in the size of the PS microsphere, the *R*_ct_ decreased, and the arcs of PS350-SnO_2_, PS480-SnO_2_, and PS600-SnO_2_ perovskite solar cells gradually decreased with the increase in pore size.^28^ This further supports the evidence that the size of PS microspheres that is too much increased negatively impacts the PCE performance due to the decrease in surface area. What is more, the *R*_s_ of all the mesoporous devices are lower than those of the planar structure, indicating a decrease in series resistance, which is mainly attributed to the parallel pathway created by the individual honeycomb units and more compacted interface connections; similarly, a further increase in the initial size to 600 nm of PS microspheres increased the *R*_s_, which could be ascribed to the detriments of the morphology. Therefore, all three major issues of the macroporous framework in the morphology of honeycomb cells, which are stacked as an inverse opal structure, are illustrated.

**Table 2:** Impedance and Other Parameters for EIS for MAPbI_3_ PSCs Based on Different Inverse Electron Transport Layers

	*R*_S_ (Ω)	*R*_CT_ (Ω)	*Q*_2_[10^–6^ F·s^a^^–1^]	*a*
Flat-SnO_2_	9.524	192.3	0.1051	0.9696
PS350-SnO_2_	4.434	457.6	0.4168	0.8665
PS480-SnO_2_	4.791	433.4	0.2881	0.8909
PS600-SnO_2_	6.806	374.1	0.1627	0.9242

**Figure 5:**
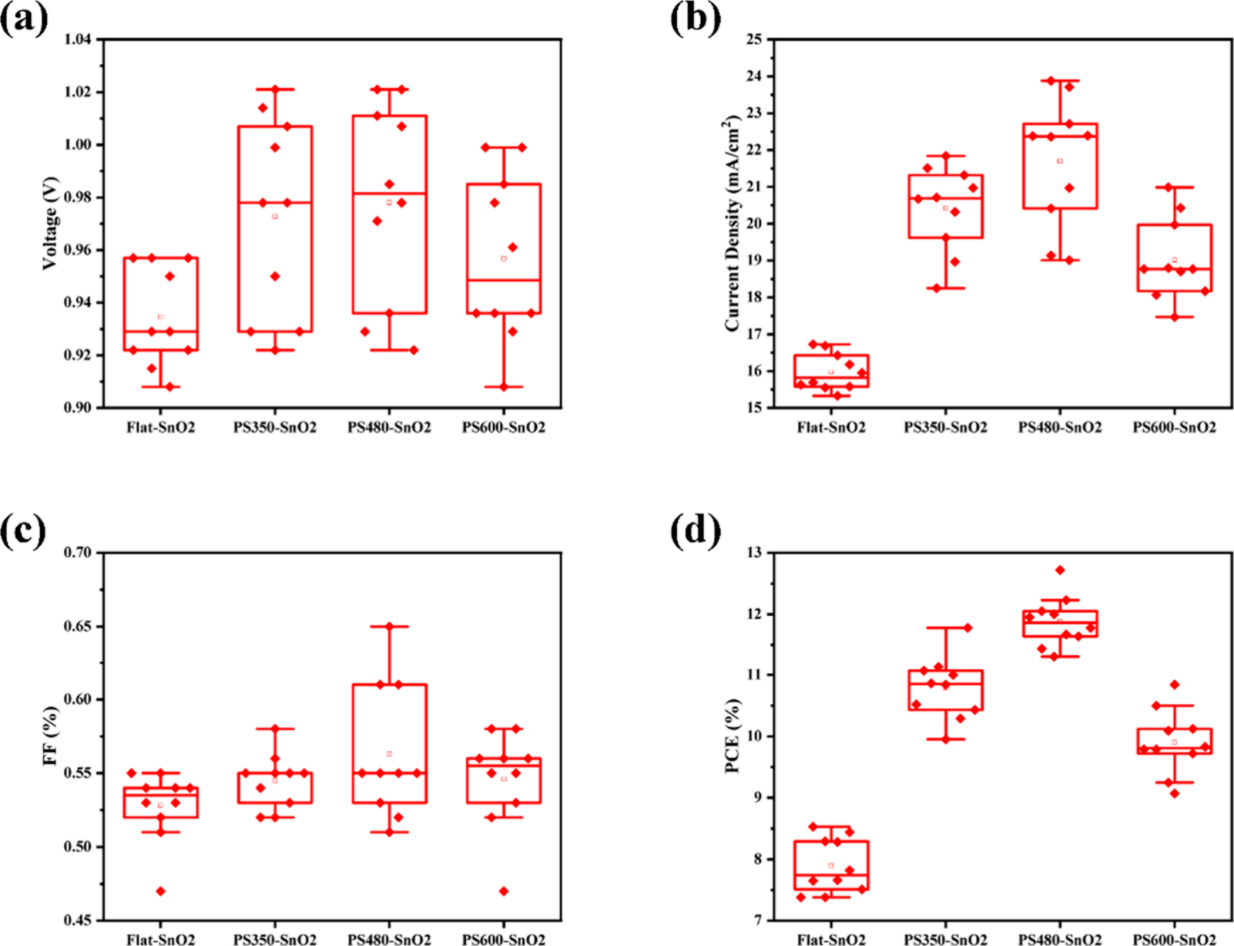
Statistical distribution of performance parameters (a) *V*_OC_, (b) *J*_SC_, (c) FF, and (d) PCE obtained from the measurement of *J*–*V* curves for MAPbI_3_ PSCs fabricated on PS350-SnO_2_, PS480-SnO_2_, PS600-SnO_2_, and Flat-SnO_2_ layers.

Figure 5 presents the statistical distribution of photovoltaic performance parameters for the groups of solar cells. Due to the energy level match between the materials used for perovskite, the electron transport layer, and the electrode, there is no significant variation in the VOC (voltage open circuit) of the group samples, as shown in Figure 5a. Among them, the PS480-SnO_2_ layer with the MAPbI_3_ perovskite shows the highest improvement of approximately 55% compared to the Flat-SnO_2_ layer. This enhancement can be clearly observed in Figure 5d. For the long-term aging investigations, Figures S3 and S4 represent the stability test of inverse perovskite solar cells conducted at room temperature with a relative humidity of 25%. The PCE continuously decreases for the first 144 h and gradually stabilizes thereafter. After 1440 h of aging, the PCE can maintain 88% of its initial value.

## 4 Conclusions

In summary, the inverse opal structure of perovskite was prepared by using a sacrificial template method. Three different pore size inverse opal layers were fabricated by vertically self-assembling polystyrene microspheres with diameters of 350, 480, and 600 nm and by controlling the spinning parameters to constrain into a single-layer framework. These layers were deposited on top of a dense electron transport layer in contact with the perovskite layer, with the covering carbon electrode as PSCs. Experimental results demonstrated that compared to the planar structure, the single-layer inverse opal structure exhibited enhanced light absorption, leading to an increased short-circuit current (*J*_SC_) and fill factor (FF) due to the increased surface area through electronic transport optimizations. Among them, the inverse opal layer prepared with 480 nm polystyrene microspheres showed superior photovoltaic performance parameters for a proper surface area and relatively increased light absorptions. Ultimately, the power conversion efficiency (PCE) of the MAPbI_3_ perovskite solar cell was improved from 7.51 to 11.67%, representing a relative enhancement of 55%, and the aging tests prove that the devices with the additional structural layer have a good endurance under a conventional atmosphere after 1440 h of aging.

## Supporting Information Available

Digital images under daylight of the inverse opal SnO_2_ and planar SnO_2_, AFM images for the PS480-SnO_2_, aging test for the PS480-SnO_2_-based perovskite solar cells, and detailed PCE aging test for the PS480-SnO_2_-based perovskite solar cells (PDF)
