# Supplementary material for: Integrating Low-Stack Photonic Crystals with the Honeycomb-like Structural Framework to Enhance the Photovoltaic Performance in Perovskite Solar Cells
Source: ACS Omega. 2024 Feb 15;9(8):9720–7. doi: 10.1021/acsomega.3c09868 (PMC10906030; doi:10.1021/acsomega.3c09868)
Supplement: Supplementary file 1 — ao3c09868_si_001.pdf [file ao3c09868_si_001.pdf]

Supporting Information

Integrating Low-Stack Photonic crystals with the Honeycomb-like Structural Framework to Enhance the Photovoltaic Performance in Perovskite Solar Cells

Chen Yuan, Yibin Yang, Le Huang, Ye Xiao⁎

School of Materials and Energy, Guangdong University of Technology,

Guangzhou 510006, China

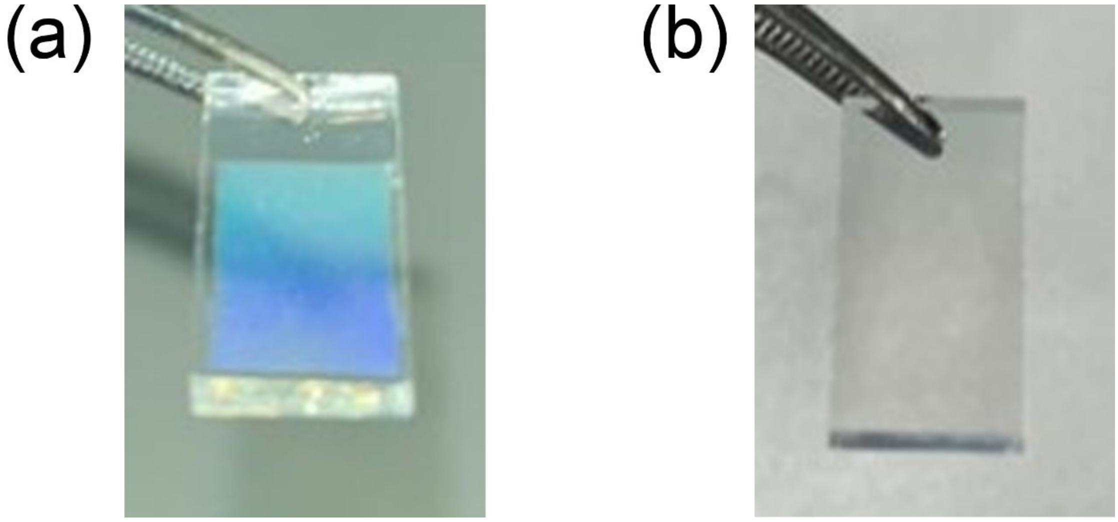

Figure S1. The digital images under daylight of the inverse opal-SnO_2 (a) and planar SnO_2.

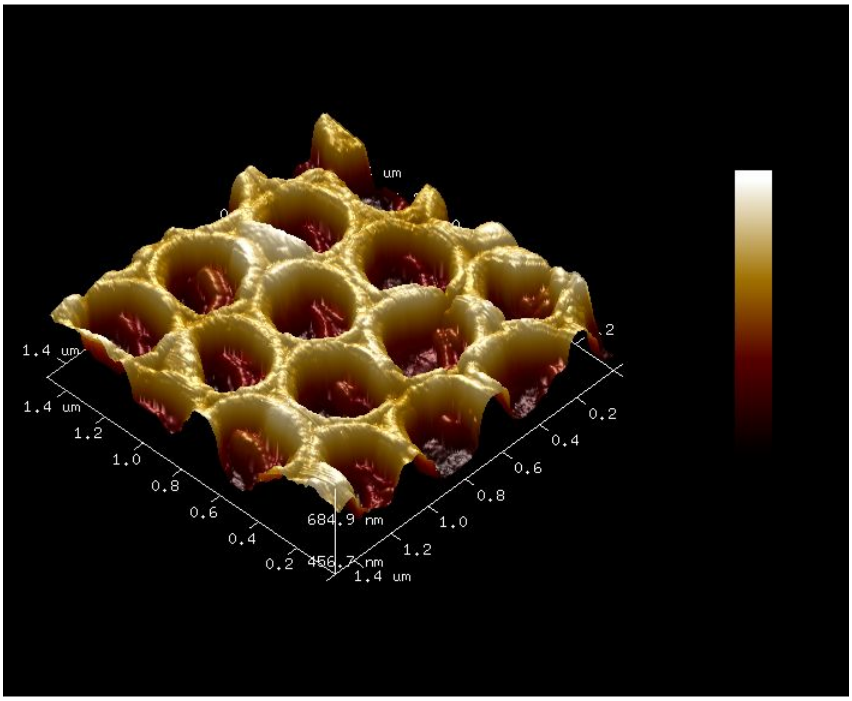

Figure S2. AFM images for the PS480-SnO_2

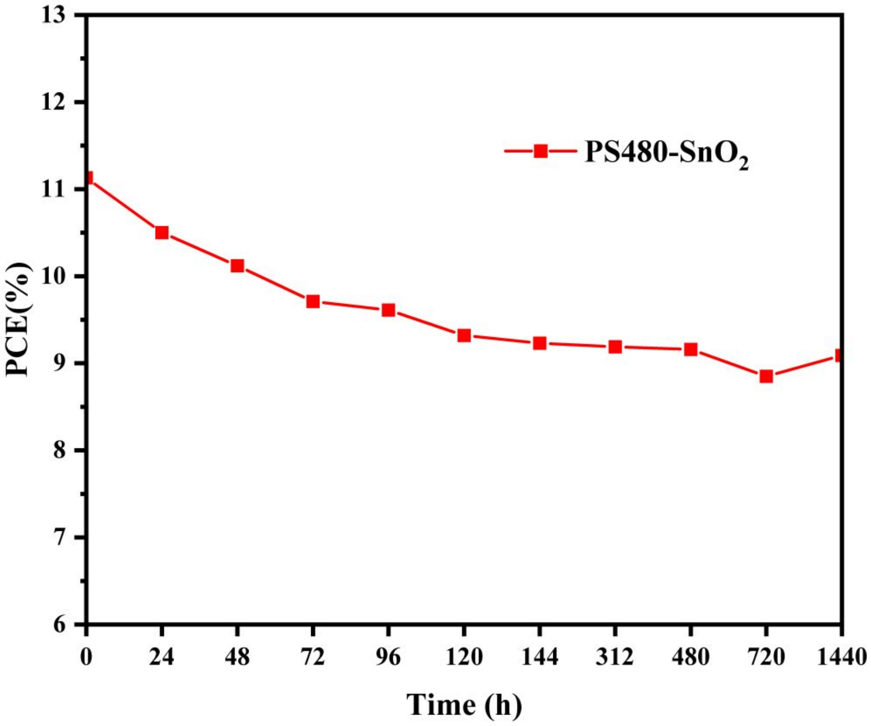

Figure S3. Aging test for the PS480-SnO_2 based perovskite solar cells.

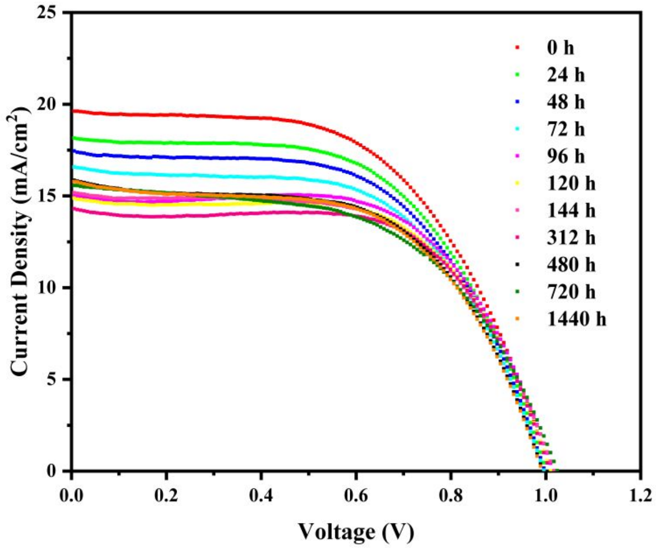

Figure S4. Detailed PCE aging test for the PS480-SnO_2 based perovskite solar cells.
